# Inhibition of PKR protects against H_2_O_2_-induced injury on neonatal cardiac myocytes by attenuating apoptosis and inflammation

**DOI:** 10.1038/srep38753

**Published:** 2016-12-08

**Authors:** Yongyi Wang, Min Men, Bo Xie, Jianggui Shan, Chengxi Wang, Jidong Liu, Hui Zheng, Wengang Yang, Song Xue, Changfa Guo

**Affiliations:** 1 Department of Cardiovascular Surgery, Ren Ji Hospital, School of Medicine, Shanghai Jiao Tong University, Shanghai, China; 2 Department of endocrinology, Xi’an Central Hospital, Shaanxi, China; 3 Department of Cardiovascular Surgery, Zhong Shan Hospital, School of Medicine, Fudan University, Shanghai, China

## Abstract

Reactive oxygenation species (ROS) generated from reperfusion results in cardiac injury through apoptosis and inflammation, while PKR has the ability to promote apoptosis and inflammation. The aim of the study was to investigate whether PKR is involved in hydrogen peroxide (H_2_O_2_) induced neonatal cardiac myocytes (NCM) injury. In our study, NCM, when exposed to H_2_O_2_, resulted in persistent activation of PKR due to NCM endogenous RNA. Inhibition of PKR by 2-aminopurine (2-AP) or siRNA protected against H_2_O_2_ induced apoptosis and injury. To elucidate the mechanism, we revealed that inhibition of PKR alleviated H_2_O_2_ induced apoptosis companied by decreased caspase3/7 activity, BAX and caspase-3 expression. We also revealed that inhibition of PKR suppressed H_2_O_2_ induced NFκB pathway and NLRP3 activation. Finally, we found ADAR1 mRNA and protein expression were both induced after H_2_O_2_ treatment through STAT-2 dependent pathway. By gain and loss of ADAR1 expression, we confirmed ADAR1 modulated PKR activity. Therefore, we concluded inhibition of PKR protected against H_2_O_2_-induced injury by attenuating apoptosis and inflammation. A self-preservation mechanism existed in NCM that ADAR1 expression is induced by H_2_O_2_ to limit PKR activation simultaneously. These findings identify a novel role for PKR/ADAR1 in myocardial reperfusion injury.

Myocardial ischemia reperfusion injury is a major mechanism leading to cell damage and organ dysfunction after myocardial infarction. A great part of cardiomyocytes cell death occurs via the process of acute reperfusion phase due to the development of oxidative stress induced by the generation of reactive oxygen species (ROS), such as H_2_O_2_^1^,^2^. H_2_O_2_ is highly diffusible and triggers subsequent inflammation, thus causing severe cardiac cell apoptosis and injury^3^.

Inflammatory injury limits the benefits of reperfusion during acute myocardial infarction. Therefore, protecting cardiomyocyte from inflammatory damage could be a rational method for ameliorating myocardial reperfusion injury^4^,^5^. NFκB and NLRP3 inflammasome pathway are both important inflammatory signaling, and new targeted therapeutic strategies such as anti-NFκB and anti-NLRP3 inflammasome were explored^6^,^7^,^8^. However, it is unclear how these inflammatory signaling molecules are coordinately regulated in myocardial reperfusion injury process. Myocardial apoptosis is one of major damage pattern which contributes to myocardial reperfusion injury^9^,^10^. Therefore, various ways to alleviate apoptosis were also extensively investigated.

Protein kinase PKR is double-stranded RNA (dsRNA)-activated serine/threonine protein kinase inducible by interferon(IFN). It was first identified as a mediator of the antiproliferative and antiviral actions of interferon. It is an ubiquitously expressed protein in mammalian cells and carry the potential ability for direct recognition of pathogens and activation of specific cellular responses to external stimuli. Encountering infections, PKR can regulate or act in conjunction with major inflammatory signaling pathways that are implicated in myocardial ischemia reperfusion injury, including NFκB and inflammasome NLRP3^11^,^12^,^13^,^14^. In addition to regulating inflammatory signaling, PKR also promotes apoptosis through interactions with FAS-associated death domain protein and upregulation of the proapoptotic factor BAX^15^,^16^. Taken together, PKR is a potential target for treatment of myocardial reperfusion injury. Accumulating evidences also revealed that inflammatory stressors such as TNF-α, lipopolysaccharide (LPS), viral dsRNA and its mimetic compound poly (I:C), all activate the RNA editor ADAR1^17^,^18^,^19^. By interacting with PKR and inhibiting its kinase activity, ADAR1 presents a role in buffering inflammatory stress effects^18^. Therefore, it is possible that ADAR1 also modulate PKR activity in cardiac myocytes.

In the present study, we examined the role of PKR in H_2_O_2_-induced injury on NCM. We observed that PKR was activated by endogenous RNA from H_2_O_2_ treated NCM, and inhibition of PKR activation significantly decreased H_2_O_2_ induced injury. The mechanisms involve the suppression of NFκB, NLRP3 inflammasome activation, and alleviation of apoptosis. We also confirmed ADAR1 was up-regulated after H_2_O_2_ treatment, and ADAR1 modulated PKR activity.

## Results

### H_2_O_2_ induces PKR phosphorylation in NCM

**Figure 1:**
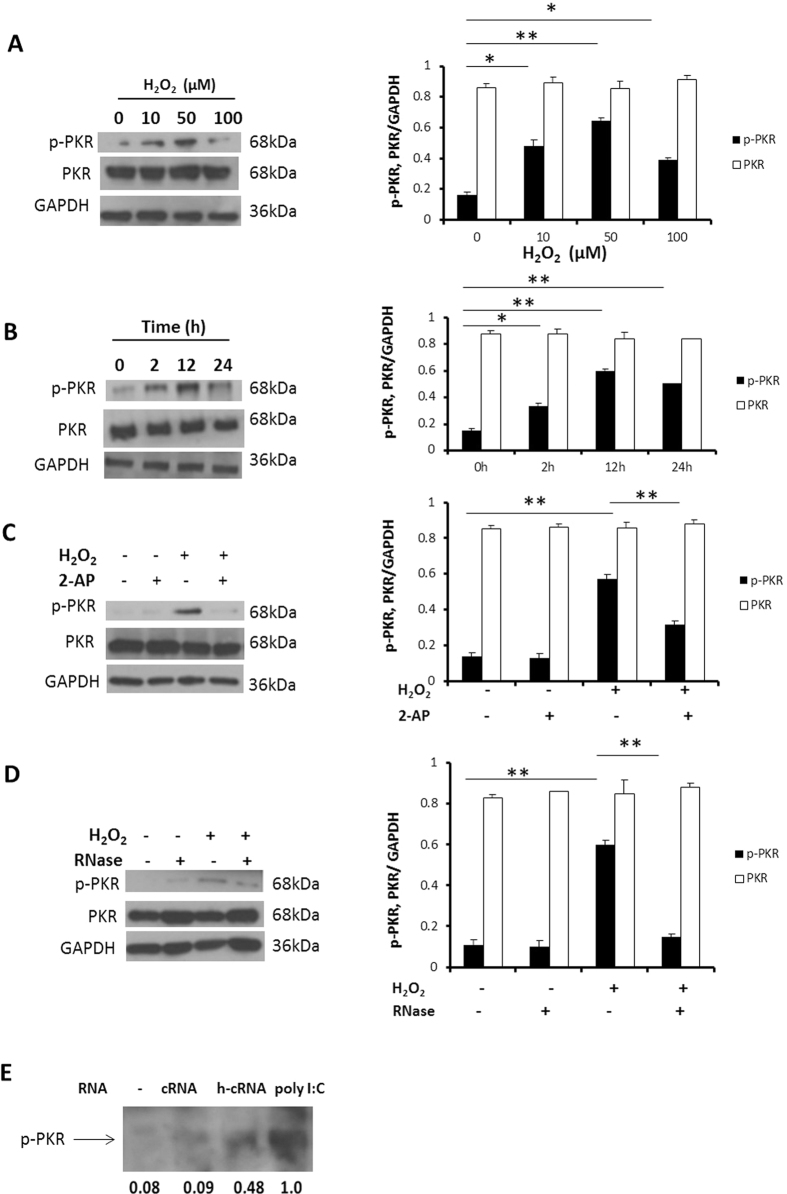
H_2_O_2_ induces PKR phosphorylation in NCM. (**A**) Cultured NCM were treated with H_2_O_2_ (0–100 μM) for 12 h. (**B**) Cultured NCM were treated with H_2_O_2_ (50 μM) for 24 h. (**C**) Cultured NCM were left untreated or stimulated with 4 mM 2-AP for 30 minutes, followed by stimulation with H_2_O_2_ (50 μM) for 12 h. (**D**) Cultured NCM were left untreated or stimulated with 100 μg/ml RNase for 30 minutes, followed by stimulation with H_2_O_2_ (50 μM) for 12 h. (**A,B,C,D**) The expression of p-PKR, PKR protein were measured by Western blotting, densitometric analyses of p-PKR, PKR/GAPDH were calculated. (**E**) PKR was from NCM and subjected to PKR binding assay. Purified PKR was incubated without or with 1 μg/ml of total RNA from NCM, NCM treated with H_2_O_2_ (50 μM) for 12 h, or Poly I:C. The data are representative of three independent experiments. (**P < 0.01; *P < 0.05). Full length blots were shown in Supple Fig. 4.

**Figure 2:**
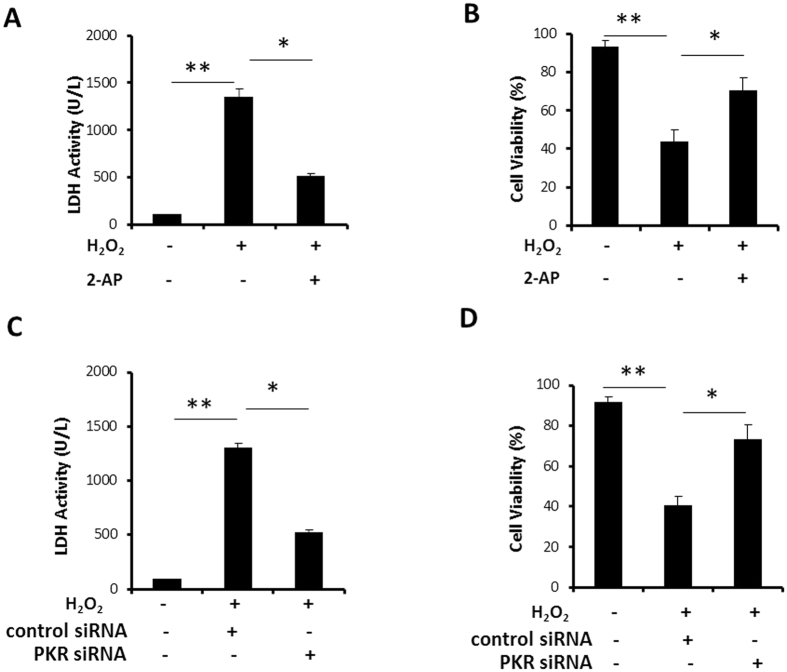
Inhibition of PKR alleviates H_2_O_2_ induced NCM injury. (**A,B**) Cultured NCM were left untreated or stimulated with 4 mM 2-AP for 30 minutes, followed by stimulation with H_2_O_2_ (50 μM) for 24 h. (**C,D**) Cultured NCM were left untreated or transfected with siRNA, followed by stimulation with H_2_O_2_ (50 μM) for 24 h. (**A,C**) Cultured NCM media were collected for LDH determination. (**B,D**) Cultured NCM were collected for cell viability determination. The data are representative of three independent experiments. (**P < 0.01; *P < 0.05).

We first examined whether H_2_O_2_ was able to activate PKR in NCM. As shown in Fig. 1A, 12 h exposure of NCM to H_2_O_2_ resulted in the increased PKR phosphorylation. H_2_O_2_-mediated increase in PKR phosphorylation is in a dose-dependent manner with a peak at approximately 50 μM. Because the maximum induction of PKR phosphorylation occurred at the concentration of 50 μM, all subsequent experiments were conducted according to this concentration. Due to the key role of oxidative stress in PKR activation, we also determined the levels of ROS after 50 μM H_2_O_2_ stimulation. We found H_2_O_2_ treatment resulted in maximum elevation of ROS after 2 h, and sustained 24 h (Supple Fig. 1). Next, NCM were stimulated with H_2_O_2_ (50 μM) for 0, 2, 12, 24 hours and PKR phosphorylation was evaluated. PKR was found to be activated by H_2_O_2_ after 2 h, reaching maximum phosphorylation levels at 12 h (Fig. 1B). 2-AP was extensively used as a PKR activation inhibitor. We confirmed 4 mM is the minimum concentration to inhibit PKR activation in H_2_O_2_ treated NCM (Supple Fig. 2). Pretreatment of NCM with the 2-AP for 30 minutes before stimulation with the H_2_O_2_ significantly suppressed PKR phosphorylation (Fig. 1C). PKR is a key component of the cytoplasmic RNA sensors involved in the recognition of viral double-stranded RNA (dsRNA), and bacterial RNAs can directly binds to and activate PKR in human adult cardiac myocytes^20^. Therefore, we tested whether endogenous RNA released from injured NCM can activate PKR. We found RNase abolished H_2_O_2_ induced PKR activation completely (Fig. 1D). It reveals endogenous RNA results in phosphorylation of PKR. Then, we performed the PKR binding assay to elucidate the mechanism. We found RNA from untreated NCM was unable to activate PKR, however, RNA from H_2_O_2_ treated NCM resulted in phosphorylation of PKR. As a positive control, synthetic dsRNA (poly I:C) activated PKR distinctly (Fig. 1E).

### Inhibition of PKR alleviates H_2_O_2_ induced NCM injury

**Figure 3:**
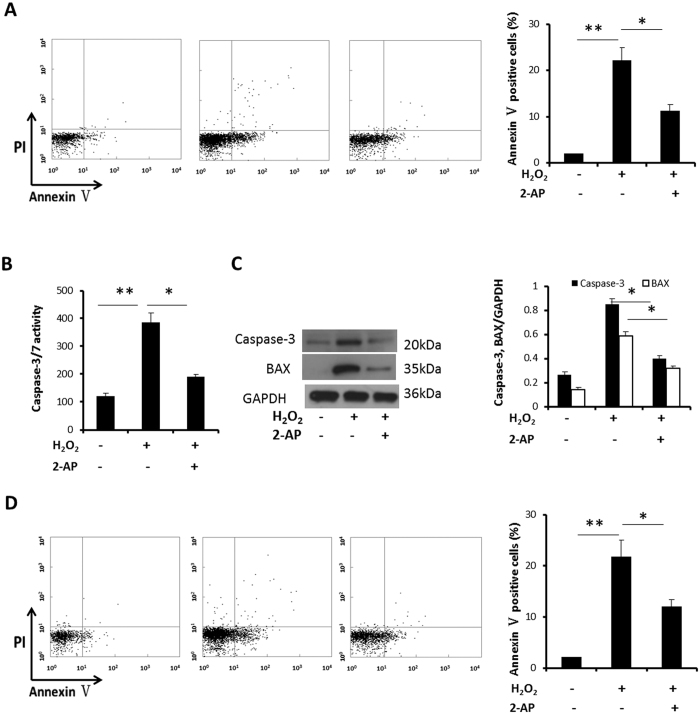
H_2_O_2_ induced NCM apoptosis is PKR dependent. (**A,B,C**) Cultured NCM were left untreated or stimulated with 4 mM 2-AP for 30 minutes, followed by stimulation with H_2_O_2_ (50 μM) for 12 h. (**A**) Analysis of NCM apoptosis by flow cytometry after staining cells with Annexin V and PI. Percentage of Annexin V positive cells were calculated. (**B**) Related enzyme activity of NCM apoptosis. (**C**) The expression of BAX, caspase-3 protein were measured by Western blotting, and densitometric analyses of BAX, caspase-3/GAPDH were calculated. The data are representative of three independent experiments. (**D**) Cultured NCM were left untreated or transfected with siRNA, followed by stimulation with H_2_O_2_ (50 μM) for 12 h. Analysis of NCM apoptosis by flow cytometry after staining cells with Annexin V and PI. Percentage of Annexin V positive cells were calculated. (**P < 0.01; *P < 0.05). Full length blots were shown in Supple Fig. 4.

The integrity of cell membranes or necrosis is estimated by LDH release into the media in response to oxidant burden. Figure 2A shows H_2_O_2_ induced LDH activity in NCM significantly reduced by 2-AP (1483.77 ± 27.60 vs 489.06 ± 16.74, P = 0.03). Figure 2B shows H_2_O_2_ markedly decreased cell viability, compared with untreated cells (93.43 ± 3.10 vs 43.96 ± 6.26, P = 0.002). However, H_2_O_2_ induced decrease of cell viability was significantly attenuated by 2-AP pretreatment (43.96 ± 6.26 vs 70.45 ± 7.08, P = 0.02). To exclude the possibility that 2-AP may affect the NCM survival independent to its effect of PKR inhibition, and further confirm the role of PKR in alleviating H_2_O_2_ induced NCM injury, NCM were transfected with siRNA for PKR. First, we confirmed the knockdown efficiency of PKR by Western blotting (Supple Fig. 3). Figure 2C shows H_2_O_2_ induced LDH activity in NCM obviously reduced after down regulation of PKR (1306.95 ± 36.31 vs 520.43 ± 25.28, P = 0.03). Figure 2D reveals H_2_O_2_ induced decrease of cell viability was significantly attenuated by PKR siRNA (41.45 ± 4.74 vs 73.11 ± 7.27, P = 0.02).

### H_2_O_2_ induced NCM apoptosis is PKR dependent

PKR mediates transverse aortic constriction (TAC) induced cardiac myocytes apoptosis^21^. Therefore, NCM apoptosis was assessed after H_2_O_2_ treatment with (without) 2-AP pretreatment. We observed NCM apoptosis obviously increased when exposed to H_2_O_2_ (2.01 ± 0.12 vs 22.13 ± 2.86, P = 0.004). However, this increase attenuated significantly after 2-AP pretreatment (22.13 ± 2.86 vs 11.24 ± 1.43, P = 0.02) (Fig. 3A). Next, we examined the effect of 2-AP on H_2_O_2_ induced caspase-3/7 activity on NCM. Figure 3B shows that H_2_O_2_ induced caspase3/7 activity. However, 2-AP markedly suppressed H_2_O_2_-induced caspase-3/7 activity. To investigate the mechanism, we detected the proapoptotic factor Bax and caspase-3 expression. We found H_2_O_2_ induced Bax and caspase-3 expression, but this increase was significantly buffered after 2-AP pretreatment (Fig. 3C). These data suggest that inhibition of PKR reduced H_2_O_2_ induced apoptosis by restricting Bax and Caspase-3 expression. We also evaluated the apoptosis after PKR siRNA transfection. We demonstrated NCM apoptosis obviously increased when exposed to H_2_O_2_ (2.14 ± 0.13 vs 21.79 ± 3.23, P = 0.004), and this increase attenuated significantly after PKR siRNA pretreatment (21.79 ± 3.23 vs 12.07 ± 1.39, P = 0.02) (Fig. 3D). It revealed 2-AP didn’t affect the NCM apoptosis.

### PKR mediates NFκB activation signaling in H_2_O_2_ treated NCM

**Figure 4:**
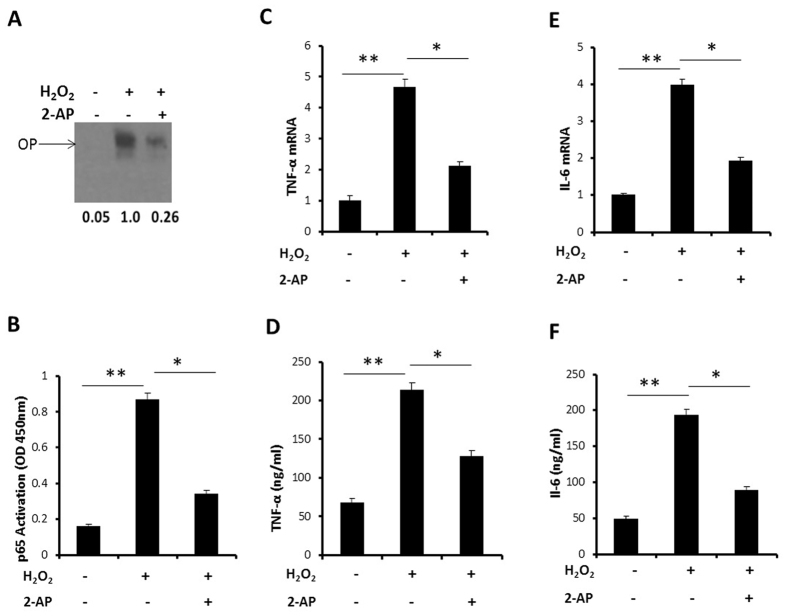
PKR mediates NFκB activation signaling in H_2_O_2_ treated NCM. Cultured NCM were left untreated or stimulated with 4 mM 2-AP for 30 minutes, followed by stimulation with H_2_O_2_ (50 μM) for 12 h. (**A**) Analysis of NFκB activation by EMSA; (**B**) Analysis of NFκB activation by TransAM p65 NFκB assay kit. (**C**) Real-time PCR analysis of TNF-α mRNA expression. (**D**) ELISA assay for detection the TNF-α release in medium. (**E**) Real-time PCR analysis of IL-6 mRNA expression. (**F**) ELISA assay for detection the IL-6 release in medium. The data are representative of three independent experiments. (**P < 0.01; *P < 0.05). Full length blots were shown in Supple Fig. 4.

We first conducted EMSA to assess whether the activation of NFκB was PKR phosphorylation dependent. We found NFκB activation increased after H_2_O_2_ treatment compared to that in untreated group. However, this increase was inhibited after pretreatment with 2-AP (Fig. 4A). Activation of the p65 subunit of NFκB was also determined with a NFκB p65 ELISA-based assay. P65 activation induced by H_2_O_2_ and was found to be significantly reduced after 2-AP pretreatment (Fig. 4B). Next, we determined the downstream inflammatory cytokines, such as TNF-α and IL-6, mRNA expression and protein release in medium. As shown in Fig. 4C,D,E,F, H_2_O_2_ induced TNF-α and IL-6 mRNA expression and protein release in medium. 2-AP pretreated NCM displayed a significantly reduced H_2_O_2_ induced TNF-α and IL-6 mRNA expression and protein release in medium. These data indicate PKR phosphorylation is essential for H_2_O_2_ induced NFκB activation signaling.

### PKR is involved in H_2_O_2_ induced inflammasome activation in NCM

**Figure 5:**
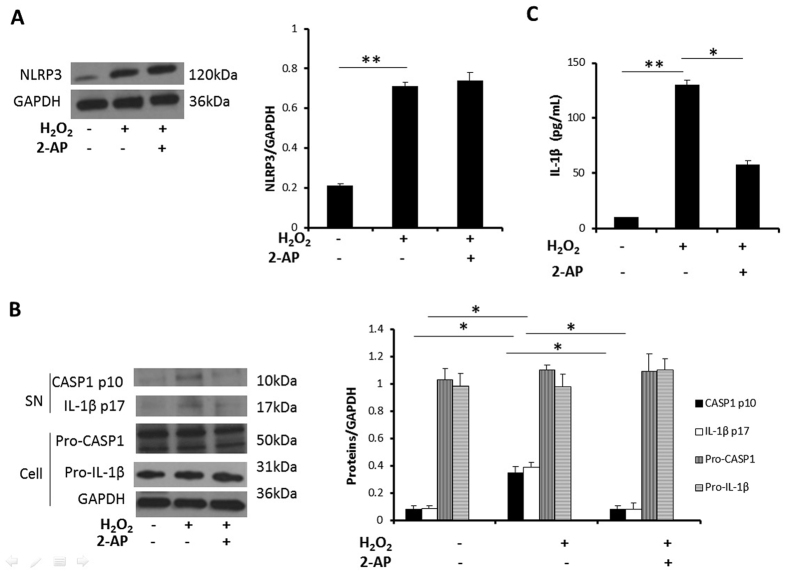
PKR is involved in H_2_O_2_ induced inflammasome activation in NCM. Cultured NCM were left untreated or stimulated with 4 mM 2-AP for 30 minutes, followed by stimulation with H_2_O_2_ (50 μM) for 12 h. (**A**) The expression of NLRP3 was measured by Western blotting, and densitometric analysis of NLRP3/GAPDH was calculated. (**B**) The expression of CASP1 p10, IL-1β p17, Pro-CASP1 and Pro-IL-1β proteins were measured by Western blotting, and densitometric analyses of CASP1 p10, IL-1β p17, Pro-CASP1 and Pro-IL-1β/GAPDH were calculated. (**C**) ELISA assay for detection the IL-1β release in medium. (**P < 0.01; *P < 0.05). Full length blots were shown in Supple Fig. 4.

PKR mediates inflammasome activation by physically interacting with NLRP3 in mice macrophages^12^. In this study, we found NLRP3 expression increased obviously after H_2_O_2_ treatment. However, the expression of NLRP3 did not differ significantly after 2-AP pretreatment in NCM (Fig. 5A). Next, we determined caspase-1 activation and IL-1β cleavage in NCM. Though the expressions of pro-IL-1β and pro-CASP1 were stable, caspase-1 activation and IL-1β cleavage in NCM both increased after exposed to H_2_O_2_ and inhibited after 2-AP pretreatment (Fig. 5B). We also measured IL-1β release in medium. As expected, IL-1β release increased after H_2_O_2_ treatment, and attenuated after 2-AP pretreatment (Fig. 5C).

### ADAR1 is induced by H_2_O_2_ and modulates PKR activation

**Figure 6:**
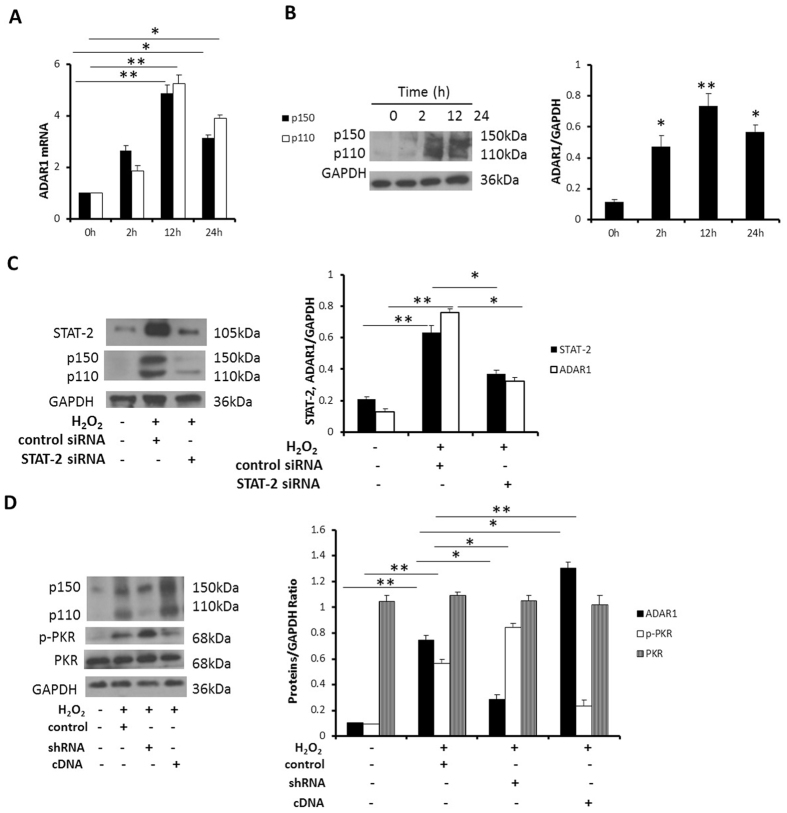
ADAR1 is induced by H_2_O_2_ and modulates PKR activation. (**A**) Cultured NCM were treated with H_2_O_2_ (50 μM) for 24 h. Real-time PCR analysis of p150, p110 mRNA expression. (**B**) Cultured NCM were treated with H_2_O_2_ (50 μM) for 24 h. The expression of ADAR1 protein was measured by Western blotting, and densitometric analysis of ADAR1/GAPDH was calculated. (**C**) Cultured NCM were transfected with STAT-2 siRNA, followed by H_2_O_2_ (50 μM) for 12 h. The expression of STAT-2, ADAR1 protein were measured by Western blotting, and densitometric analyses of STAT-2, ADAR1/GAPDH were calculated. The data are representative of three independent experiments. (**D**) Cultured NCM were transfected with adenovirus containingADAR1-specific shRNA or ADAR1 cDNA for the purpose of knocking down or over expressing ADAR1, followed by H_2_O_2_ (50 μM) for 12 h. The expression of ADAR1, p-PKR and PKR protein were measured by Western blotting, and densitometric analyses of ADAR1, p-PKR and PKR/GAPDH were calculated. The data are representative of three independent experiments. (**P < 0.01; *P < 0.05). Full length blots were shown in Supple Fig. 4.

Previous reports revealed ADAR1 might affect PKR autophosphorylation^18^,^22^ We first examined changes of ADAR1 expression in NCM after H_2_O_2_ treatment. Both ADAR1 mRNA and protein levels were significantly increased with a peak in 12 hours (Fig. 6A,B). Next, we tested how ADAR1 expression was manipulated. After downregulation of STAT-2 by siRNA, we found ADAR1 expression was suppressed (Fig. 6C). Finally, we tested the effect of ADAR1 on the status of PKR phosphorylation in NCM. We observed though the total PKR Levels were unchanged, the phosphorylated PKR was downregulated while overexpression of ADAR1. We also confirmed that ADAR1 knockdown in NCM exposed to H_2_O_2_ resulted in significantly increased PKR activation with unaffected total PKR levels (Fig. 6D). These data consistently indicated that ADAR1 modulated PKR activation in NCM.

## Discussion

In addition to its antiviral function, it is noteworthy that PKR responds to various cellular stresses^23^,^24^ and regulates cell inflammation, proliferation^25^, and apoptosis. However, most of the previous studies revealed the roles of PKR in confronting the chronic cellular stresses, including regulating metabolic homeostasis^23^, mediating TNF-α induced osteoclast formation^26^ and systolic overload-induced congestive heart failure^21^, *et al*. In this study, we investigated in the face of H_2_O_2_ treatment on NCM, an acute and drastic cellular stress, whether PKR is involved. We found consistently activation of PKR after H_2_O_2_ treatment, and we also revealed PKR inhibition protected against H_2_O_2_-induced injury by attenuating apoptosis and inflammation.

PKR is a key component of the cytoplasmic RNA sensors involved in the recognition of viral double-stranded RNA (dsRNA). Accumulating evidences suggested specific nucleoside modifications and structural elements are differentially represented in either microbial or mammalian RNA and therefore provide a molecular mechanism to discriminate foreign from self RNA^27^,^28^. However, under some situations, self cellular primary transcripts undergo some modifications and are recognized. The RNA released from necrotic cells may act as an endogenous TLR3 ligand for the stimulation of proinflammatory gene expression in rheumatoid arthritis synovial fibroblasts^29^. It has been described that endogenous RNA with a 5′-triphosphate-dependent manner can interact with PKR after induction of metabolic stress by palmitic acid (PA). This study by Youssef OA *et al*. indicated snoRNAs were enriched in response to PA and a subset of identified snoRNAs could bind and activate PKR *in vitro*^30^. In this study, we found that RNA from normal NCM was unable to activate PKR. However, RNA from H_2_O_2_ treated NCM resulted in PKR phosphorylation directly. The following study was warranted to identify the particular types of endogenous RNA released from H_2_O_2_ treated NCM.

NFκB, known as the major transcriptional factor of a wide range of inflammatory cytokines, triggered inflammatory response via modulating inflammatory transcription and in turn maintained NFκB activation and establish a positive autoregulatory loop to sustain the inflammatory status^31^. The role of NFκB activation and their downstream cytokines, such as TNF-α, in myocardial reperfusion injury has been previously reported, that myocardial ischemia reperfusion injury was alleviated after NFκB inhibition^7^,^32^. PKR has the property to mediate inflammatory signaling through NFκB activation. As expected, in our study, inhibition of PKR activation suppressed NFκB activation and downstream inflammatory cytokines expression. One of the recently identified proinflammatory signaling pathways involved in myocardial ischemia reperfusion injury is NLRP3 inflammasome, that inhibition of NLRP3 activation limited inflammatory injury following myocardial ischemia reperfusion^6^. PKR physically interacts with NLRP3 and mediates cleavage of inactive pro- IL-1β to their active form in mouse macrophage^12^. In our study, though NLRP3 expression was induced after H_2_O_2_ treatment, NLRP3 expression was stable after 2-AP pretreatment. It indicates NLRP3 expression is not PKR activation dependent. However, IL-1β cleavage and caspase-1 activation were significantly inhibited in NCM by exposure to H_2_O_2_ after 2-AP treatment, indicating that inhibition of PKR activation abrogates NLRP3 inflammasome activation. Taken together, inhibition of PKR alleviated the inflammation by suppressing the NFκB pathway and NLRP3 inflammasome activation, which were both involved in reperfusion induced injury. We deduce alleviation of inflammation after inhibition of PKR activation provided the cardiac protection against H_2_O_2_.

Loss of cardiac myocytes by apoptosis is a serious and frequent complication from reperfusion injury^33^. It is well known that PKR can mediate different forms of stress-induced apoptosis through eIF2α phosphorylation^34^ and through a Fas-associated death domain protein/caspase-8/caspase-3 signaling pathway^15^. NFκB activation by PKR has been suggested to mediate apoptosis^35^,^36^. The expressions of Bax, p53 and Bcl-2 were all regulated by PKR^16^. In present study, cardiomyocytes apoptosis induced by H_2_O_2_ attenuated after PKR inhibition, accompanied by low BAX and caspase-3 expression. Though the definitive mechanism is still unknown, we found cardiomyocytes apoptosis was suppressed after PKR inhibition.

ADAR1 expression has been broadly implicated in the other inflammatory conditions^17^,^18^,^19^,^37^. In present study, we observed ADAR1 expression was induced after H_2_O_2_ treatment in NCM. Previous reports revealed a STAT-2 dependent process of transcriptional activation of IFN-induced ADAR1 expression in mouse embryo fibroblast cells^38^. In our study, we confirmed H_2_O_2_ induced ADAR1 expression in NCM was also STAT-2 dependent. The expression of the mouse ADAR1 gene found on chromosome 3F2 likewise involves the utilization of multiple promoters^39^. The alternative signaling pathways which were involved in cardiac reperfusion injury include p38, STAT3 and interferon regulatory factor 3^40^,^41^. Therefore, we can’t exclude the possibility that other signaling pathway was also involved in H_2_O_2_ induced ADAR1 expression process. In this study, we also observed over-expression of ADAR1 inhibited PKR activation. It revealed the self-preservation mechanism existed in NCM to prevent an excessive apoptosis and inflammation. Though PKR activation contributed to cardiac myocytes injury, ADAR1 expression was induced to limit PKR activation simultaneously. A similar pattern for ADAR1 in buffering inflammatory stress response in murine myoblasts was described previously^18^.

**Figure 7:**
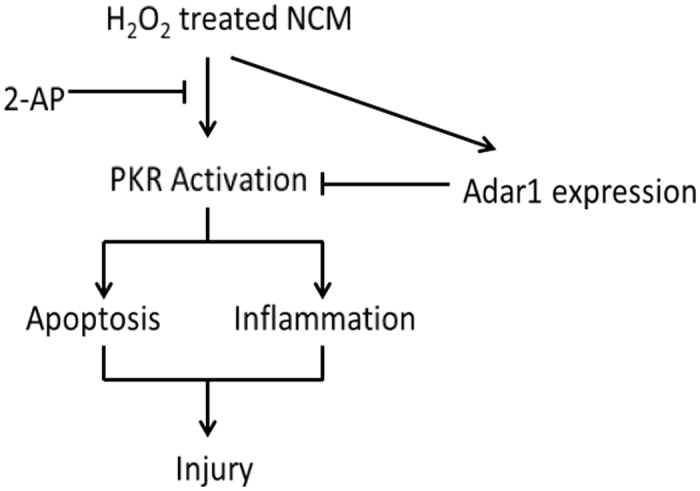
Diagram of underlying mechanism.

In conclusion, we observed PKR activation was induced by H_2_O_2_ in NCM and inhibition of PKR protects against H_2_O_2_-induced injury by attenuating apoptosis and inflammation. A self-preservation mechanism existed in cardiac myocytes that ADAR1 expression is induced by H_2_O_2_ to limit PKR activation simultaneously. The underlying mechanism is summarized in Fig. 7. These findings identify a novel role for PKR/ADAR1 in cardiac reperfusion injury.

## Materials and Methods

### NCM isolation and culture

Standard principles of laboratory animal care were followed. Male C57BL⁄6 mice (aged 6–8 wk, 18–21 g) were purchased from Shanghai Laboratory Animal Center (Shanghai, China). The present study was performed in accordance with the Guide for the Care and Use of Laboratory Animals prepared by the National Academy of Sciences, published by the National Institutes of Health. All animal procedures carried out in this study were reviewed, approved, and supervised by the Institutional Animal Care and Use Committee of Shanghai Jiao Tong University. A breeding program was performed to produce neonates. Ventricular myocardial tissues from C57BL⁄6 mice born within 24 h were minced in a nominally Ca^2+^- and Mg^2+^-free Hanks’ balanced solution. Cardiomyocytes were dispersed using 0.625 mg/mL collagenase (type II) at 37 °C for 40 min. The isolated cells were preplated for 90 min to remove non-cardiomyocytes. The cardiomyocytes were plated in M199 medium containing 10% fetal calf serum in 35 mm Petri dishes precoated with 1% gelatin. The cells were incubated at 37 °C in a humidified atmosphere containing 5% CO_2_. After the cardiomyocytes were cultured for 48 h, they were plated in dishes for the following studies. Cardiomyocytes were attached to the cell culture dishes (with approximately 70–80% confluency) and started to contract spontaneously. H_2_O_2_ (0 to 100 μM) was incubated with the cells according the experiment designs. 2-AP (Sigma) (4 mM) was dissolved in medium and incubated with cells 30 min before the addition of H_2_O_2_ (50 μM) to the cultures. RNase (Sigma) (100 μg/ml) was dissolved in medium and incubated with cells 30 min before the addition of H_2_O_2_ (50 μM) to the cultures.

### Measurement of intracellular ROS

Equal numbers of cells (10000/well in 96-well plates in Hanks Balanced Salt Solution) were treated with 10 μmol/L 2,7-dichlorofluoroscein diacetate (DCF-DA) for 3 h. Cells were washed with phosphate-buffered saline and treated with 50 μM HO during different time intervals. DCF-DA penetrates into viable cells, and inside the cells, it is converted to DCF, which later reacts with ROS and fluorescence. At the indicated time intervals, the intensity of the fluorescence was measured at an excitation wavelength of 485 nm and an wavelength of 527 nm and is expressed as percent of control fluorescence.

### Small interfering RNA (siRNA) transfection

For transient silencing of PKR or STAT-2, NCMs were transfected with mouse PKR siRNA or STAT-2 siRNA (Santa Cruz) using the siRNA Transfection Regent 24 or 48 h before following experiments. Scrambled siRNA (Santa Cruz) was also transfected as a negative control.

### Western blot

Proteins from cell-free supernatants were extracted by methanol/chloroform precipitation as described previously^42^. Cell extracts were also prepared. Samples were separated on SDS-polyacrylamide gels and electrotransferred onto nitrocellulose membranes. The membranes were blocked with 3% bovine serum albumin for 2 h and then incubated overnight at 4 °C with antibodies specific for phosphor-Thr466 on PKR (Abcam), total PKR (Abcam), caspase-3 (Cell Signaling), BAX (Abcam), IL-1β (Santa Cruz), caspase-1 (Santa Cruz), NLRP3 (Adipogen), STAT-2 (Santa Cruz), ADAR1 (Santa Cruz) and GAPDH (Sigma) followed by incubation with HRP-conjugated secondary antibody. The quantitative relative expression of proteins were calculated by normalizing the densitometric analysis to GAPDH.

### RNA preparation

Cultured cells were mock treated or 50 μM H_2_O_2_-treated for 12 h. RNA was extracted with TRIzol, followed by DNase treatment to eliminate any traces of genomic DNA contamination. RNA precipitations were collected after ethanol treatment. RNA was quantified by UV spectrometry and electrophorsed on a 1% agarose gel to verify purity and integrity prior to use.

### PKR binding assay

Cultured cells were washed with cold PBS and resuspended in RIPA lysis buffer. Then cellular lysates were precleared with protein G-Sepharose beads (Santa Cruz) and incubated with anti-PKR polyclonal antibody. The beads were washed with RIPA, collected. Immunoprecipitated PKR was incubated in kinase buffer containing 50 mM Tris-HCl, PH 7.5, 2 mM MgCl_2_, 2 mM MnCl_2_, 50 mM KCl, 0.1 mM EDTA, 1 μg/mL aprotinin, 1 mM DTT, 20% glycerol, 10 μCi (γ-^32^P) and 1 μg/mL RNA (or polyI:C). The beads were washed with salt buffer and the reaction was terminated by addition of SDS sample buffer and loaded on a 10% SDS-PAGE gel.

### Real-time PCR

Total RNA was isolated from cells using the RNeasy Mini Kit (Qiagen). Total RNA (2.5 μg) was reverse transcribed to cDNA with the SuperScript II RT kit (GIBCO BRL). mRNA expressions were determined by real time PCR using SYBR Premix Ex TaqTM (Takara, Tokoyo, Japan). The primers used for real-time PCR were as follows: IL-6, sense 5′- tgg cta agg acc aag ac cat cca a-3′, antisense 5′- aac gca cta ggt ttg ccg agt aga -3′; TNF-α, sense 5′- ctg tga agg gaa tgg gtg tt -3′, antisense 5′- ccc agc atc ttg gtt tctg -3′; ADAR1 p150, sense 5′- ggc act atg tct caa ggg ttc -3′, antisense 5′- gct gaa gct gga aac tcc tag -3′; ADAR1 p110, sense 5′- ctg aag gtg gaa gac tag gc -3′, antisense 5′- gtc caa gta cga ctg tgt ctg -3′; GAPDH 5′- cac ttg aag ggt gga gc -3′, antisense 5′- ggg cta agc agt tgg tg-3′. Data were collected and quantitatively analyzed on an ABI PRISM 7900HT sequence detection system (Applied Biosystems, Warrington, Cheshire, UK). All data were normalized automatically using GAPDH as the loading control.

### LDH measurement

The LDH content was detected using a chromatometry assay kit using a commercially available detection kit (Nanjing Jiancheng Biochemical Reagent Co., Nanjing, China) according to the manufacturer’s instructions. The absorbance of supernatant was measured at 532 nm.

### Cell viability determination

Cell viability was evaluated by the ability to reduce MTT, which is an indicator of metabolic activity. This viability assay was conducted in 96-well plates; MTT reduction was determined by spectrophotometry using a microplate reader (Bio-Tek Instruments, USA).

### Cell apoptosis analysis

The cells were harvested, washed and incubated with the solution of Annexin V-FITC for 20 min and then PI (50 μg/mL) for 10 min. All staining operations must be carried out on ice and in dark. The cells were analyzed by flow cytometer.

### Caspase3/7 activity analysis

Caspase3/7 intracellular activity was detected by Caspase-3/7 Assay Kit (Promega), and cell fluorescence intensity at 499 nm was measured by ELISA Tablet counter for quantitative assessment.

### Preparation of ADAR1 adenoviruses and virus transfection

Mouse ADAR1 cDNA (NM_001146296) and ADAR1 shRNA (sequence: gcc aag aac tac ttc aag aaa) were cloned into Pme1/Xho1 sites of a pENTCMV vector and recombined with the Ad5 backbone for virus preparation through a commercial company (Welgen, Inc. Worcester, MA). Control virus was purchased from same company. Equal amounts of adenovirus were added to cells and incubated for 24 h. The following experiments were conducted as designed.

### Electrophoretic mobility shift assay (EMSA)

The nuclear extracts (20 μg) prepared as described previously^43^ were pre-incubated for 10 min in binding buffer (1 μg poly dI-dC, 10 mmol/L Tris-HCl (pH 7.5), 50 mmol/L NaCl, 1 mmol/L EDTA, 5% glycerol, 1 mmol/L DTT, and 1 μg/μL BSA) on ice, followed by 30 min of incubation at room temperature with 1 × 10^5^ dpm (approximately 0.5 ng) of a γ-^32^P-labeled probe (Amersham) containing the NFκB binding site 5′-agt tga ggg gac ttt ccc agg c-3′ (Santa Cruz). DNA-protein complexes were run on a 6% polyacrylamide gel.

### TransAM assay

Whole cell extracts (5 μg) was used to detect activation of the p65 subunit of NFκB by the commercially available TransAM p65 NFκB assay kit (Active Motif) following the instructions of the manufacturer.

### ELISA

TNF-α, IL-6 and IL-1β levels were detected in culture medium using ELISA (R&D) according to the manufacturer’s instructions.

### Statistical analyses

All statistical analyses were performed using SPSS (version 17.0, SPSS Inc.). Values were reported as the mean ± SD. Samples were analyzed by two-tailed unpaired Student’s t-test and one-way analysis of variance (ANOVA) when appropriate. Statistical significance was defined as P < 0.05.

## Additional Information

**How to cite this article**: Wang, Y. *et al*. Inhibition of PKR protects against H_2_O_2_-induced injury on neonatal cardiac myocytes by attenuating apoptosis and inflammation. *Sci. Rep.* **6**, 38753; doi: 10.1038/srep38753 (2016).

**Publisher's note:** Springer Nature remains neutral with regard to jurisdictional claims in published maps and institutional affiliations.

## Supplementary Material

Supplementary Information

## References

[b1] Lefer D. J. & Granger D. N. Oxidative stress and cardiac disease. Am J Med 109, 315–23 (2000). 10996583 10.1016/s0002-9343(00)00467-8

[b2] Yellon D. M. & Hausenloy D. J. Myocardial reperfusion injury. N Engl J Med 357, 1121–1135 (2007). 17855673 10.1056/NEJMra071667

[b3] Clerk A., Kemp T. J., Zoumpoulidou G. & Sugden P. H. Cardiac myocyte gene expression profiling during H2O2-induced apoptosis. Physiol Genomics 29, 118–27 (2007). 17148688 10.1152/physiolgenomics.00168.2006

[b4] Minutoli L. . ROS-Mediated NLRP3 Inflammasome Activation in Brain, Heart, Kidney, and Testis Ischemia/Reperfusion Injury. Oxid Med Cell Longev 2016, 2183026, doi: 10.1155/2016/2183026 (2016). 27127546 PMC4835650

[b5] Neri M. . Cardiac oxidative stress and inflammatory cytokines response after myocardial infarction. Curr Vasc Pharmacol 13, 26–36 (2015). 23628007 10.2174/15701611113119990003

[b6] Toldo S. . Inhibition of the NLRP3 inflammasome limits the inflammatory injury following myocardial ischemia-reperfusion in the mouse. Int J Cardiol 209, 215–20, doi: 10.1016/j.ijcard.2016.02.043 (2016). 26896627

[b7] Shi Z., Lian A. & Zhang F. Nuclear factor-κB activation inhibitor attenuates ischemia reperfusion injury and inhibits Hmgb1 expression. Inflamm Res 63, 919–25, doi: 10.1007/s00011-014-0765-x (2014). 25209109

[b8] Quan W. . Magnesium lithospermate B reduces myocardial ischemia/reperfusion injury in rats via regulating the inflammation response. Pharm Biol 51, 1355–62, doi: 10.3109/13880209.2013.791324 (2013). 23863120

[b9] Park B. M., Cha S. A., Lee S. H. & Kim S. H. Angiotensin IV protects cardiac reperfusion injury by inhibiting apoptosis and inflammation via AT4R in rats. Peptides 79, 66–74, doi: 10.1016/j.peptides.2016.03.017 (2016). 27038740

[b10] Yamaguchi S. . Dental pulp-derived stem cell conditioned medium reduces cardiac injury following ischemia-reperfusion. Sci Rep 5, 16295, doi: 10.1038/srep16295 (2015). 26542315 PMC4635346

[b11] Lin S. S. . A role for protein kinase PKR in the mediation of Epstein-Barr virus latent membrane protein-1-induced IL-6 and IL-10 expression. Cytokine 50, 210–9, doi: 10.1016/j.cyto.2010.01.008 (2010). 20171114

[b12] Lu B. . Novel role of PKR in inflammasome activation and HMGB1 release. Nature 488, 670–4, doi: 10.1038/nature11290 (2012). 22801494 PMC4163918

[b13] Boriushkin E., Wang J. J., Li J., Bhatta M. & Zhang S. X. p58(IPK) suppresses NLRP3 inflammasome activation and IL-1β production via inhibition of PKR in macrophages. Sci Rep 6, 25013, doi: 10.1038/srep25013 (2016). 27113095 PMC4845006

[b14] Kumar A., Haque J., Lacoste J., Hiscott J. & Williams B. R. Double-stranded RNA-dependent protein kinase activates transcription factor NF-kappa B by phosphorylating I kappa B. Proc Natl Acad Sci USA 91, 6288–92 (1994). 7912826 10.1073/pnas.91.14.6288 PMC44186

[b15] von Roretz C. & Gallouzi I. E. Protein kinase RNA/FADD/caspase-8 pathway mediates the proapoptotic activity of the RNA-binding protein human antigen R (HuR). J Biol Chem 285, 16806–13, doi: 10.1074/jbc.M109.087320 (2010). 20353946 PMC2878037

[b16] Balachandran S. . Activation of the dsRNA-dependent protein kinase, PKR, induces apoptosis through FADD-mediated death signaling. EMBO J 17, 6888–902 (1998). 9843495 10.1093/emboj/17.23.6888 PMC1171037

[b17] Wu Y. . Adenosine deaminase that acts on RNA 1 p150 in alveolar macrophage is involved in LPS-induced lung injury. Shock 31, 410–15, doi: 10.1097/SHK.0b013e31817c1068 (2009). 18520702

[b18] Meltzer M. . The RNA editor gene ADAR1 is induced in myoblasts by inflammatory ligands and buffers stress response. Clin Transl Sci 3, 73–80, doi: 10.1111/j.1752-8062.2010.00199.x (2010). 20590675 PMC2897727

[b19] Wang H. . ADAR1 Suppresses the Activation of Cytosolic RNA-Sensing Signaling Pathways to Protect the Liver from Ischemia/Reperfusion Injury. Sci Rep 6, 20248, doi: 10.1038/srep20248 (2016). 26832817 PMC4735287

[b20] Bleiblo F. . Bacterial RNA induces myocyte cellular dysfunction through the activation of PKR. J Thorac Dis 4, 114–25, doi: 10.3978/j.issn.2072-1439.2012.01.07 (2012). 22833816 PMC3378227

[b21] Wang H. . Double-stranded RNA-dependent protein kinase deficiency protects the heart from systolic overload-induced congestive heart failure. Circulation 129, 1397–406, doi: 10.1161/CIRCULATIONAHA.113.002209 (2014). 24463368 PMC3972332

[b22] Li Z., Wolff K. C. & Samuel C. E. RNA adenosine deaminase ADAR1 deficiency leads to increased activation of protein kinase PKR and reduced vesicular stomatitis virus growth following interferon treatment. Virology 396, 316–22, doi: 10.1016/j.virol.2009.10.026 (2010). 19913273 PMC2789878

[b23] Nakamura T. . Double-stranded RNA-dependent protein kinase links pathogen sensing with stress and metabolic homeostasis. Cell 140, 338–48, doi: 10.1016/j.cell.2010.01.001 (2010). 20144759 PMC2820414

[b24] Cabanski M. . PKR regulates TLR2/TLR4-dependent signaling in murine alveolar macrophages. Am J Respir Cell Mol Biol 38, 26–31 (2008). 17690330 10.1165/rcmb.2007-0010OC

[b25] Liu X. . PKR regulates proliferation, differentiation, and survival of murine hematopoietic stem/progenitor cells. Blood 121, 3364–74, doi: 10.1182/blood-2012-09-456400 (2013). 23403623 PMC3637012

[b26] Shinohara H. . Double Stranded RNA-Dependent Protein Kinase is Necessary for TNF-α-Induced Osteoclast Formation *In Vitro* and *In Vivo*. J Cell Biochem 116, 1957–67, doi: 10.1002/jcb.25151 (2015). 25739386

[b27] Heil F. . Species-specific recognition of single-stranded RNA via toll-like receptor 7 and 8. Science 303, 1526–9 (2004). 14976262 10.1126/science.1093620

[b28] Diebold S. S. . Nucleic acid agonists for Toll-like receptor 7 are defined by the presence of uridine ribonucleotides. Eur J Immunol 36, 3256–67 (2006). 17111347 10.1002/eji.200636617

[b29] Brentano F., Schorr O., Gay R. E., Gay S. & Kyburz D. RNA released from necrotic synovial fluid cells activates rheumatoid arthritis synovial fibroblasts via Toll-like receptor 3. Arthritis Rheum 52, 2656–65 (2005). 16142732 10.1002/art.21273

[b30] Youssef O. A. . Potential role for snoRNAs in PKR activation during metabolic stress. Proc Natl Acad Sci USA 112, 5023–8, doi: 10.1073/pnas.1424044112 (2015). 25848059 PMC4413318

[b31] Christman J. W., Lancaster L. H. & Blackwell T. S. Nuclear factor kappa B: a pivotal role in the systemic inflammatory response syndrome and new target for therapy. Intensive Care Med 24(11), 1131–8 (1998). 9876974 10.1007/s001340050735 PMC7094907

[b32] Gao C. . TNF-α antagonism ameliorates myocardial ischemia-reperfusion injury in mice by upregulating adiponectin. Am J Physiol Heart Circ Physiol 308, H1583–91, doi: 10.1152/ajpheart.00346.2014 (2015). 25888509

[b33] Narula J., Hajjar R. J. & Dec G. W. Apoptosis in the failing heart. Cardiol Clin 16, 691–710, ix (1998). 9891598 10.1016/s0733-8651(05)70045-x

[b34] Scheuner D. . Double-stranded RNA-dependent protein kinase phosphorylation of the alpha-subunit of eukaryotic translation initiation factor 2 mediates apoptosis. J Biol Chem. 281, 21458–68 (2006). 16717090 10.1074/jbc.M603784200

[b35] Haneji T., Hirashima K., Teramachi J. & Morimoto H. Okadaic acid activates the PKR pathway and induces apoptosis through PKR stimulation in MG63 osteoblast-like cells. Int J Oncol. 42, 1904–10, doi: 10.3892/ijo.2013.1911 (2013). 23591640 PMC3699595

[b36] Takada Y., Ichikawa H., Pataer A., Swisher S. & Aggarwal B. B. Genetic deletion of PKR abrogates TNF-induced activation of IkappaBalpha kinase, JNK, Akt and cell proliferation but potentiates p44/p42 MAPK and p38 MAPK activation. Oncogene. 26, 1201–12 (2007). 16924232 10.1038/sj.onc.1209906

[b37] Yang J. H. . Widespread inosine-containing mRNA in lymphocytes regulated by ADAR1 in response to inflammation. Immunology. 109, 15–23 (2003). 12709013 10.1046/j.1365-2567.2003.01598.x PMC1782949

[b38] George C. X. & Samuel C. E. STAT2-dependent induction of RNA adenosine deaminase ADAR1 by type I interferon differs between mouse and human cells in the requirement for STAT1. Virology. 485, 363–70, doi: 10.1016/j.virol.2015.08.001 (2015). 26335850 PMC4619148

[b39] George C. X., Wagner M. V. & Samuel C. E. Expression of interferon-inducible RNA adenosine deaminase ADAR1 during pathogen infection and mouse embryo development involves tissue-selective promoter utilization and alternative splicing. J Biol Chem. 280, 15020–8 (2005). 15677478 10.1074/jbc.M500476200

[b40] Randall R. E. & Goodbourn S. Interferons and viruses: an interplay between induction, signalling, antiviral responses and virus countermeasures. J Gen Virol. 89 (Pt 1), 1–47 (2008). 18089727 10.1099/vir.0.83391-0

[b41] Liu X. . Identification and characterization of a constitutively expressed Ctenopharyngodon idella ADAR1 splicing isoform (CiADAR1a). Dev Comp Immunol. 63, 10–7, doi: 10.1016/j.dci.2016.05.008 (2016). 27185203

[b42] Martinon F., Pétrilli V., Mayor A., Tardivel A. & Tschopp J. Gout-associated uric acid crystals activate the NALP3 inflammasome. Nature 440, 237–41 (2006). 16407889 10.1038/nature04516

[b43] Wang Y., Men M., Yang W., Zheng H. & Xue S. MiR-31 downregulation protects against cardiac ischemia/reperfusion injury by targeting protein kinase C epsilon (PKCε) directly. Cell Physiol Biochem 36, 179–90, doi: 10.1159/000374062 (2015). 25925791

