# Supplementary material for: Inhibition of PKR protects against H2O2-induced injury on neonatal cardiac myocytes by attenuating apoptosis and inflammation
Source: Sci Rep. 2016 Dec 8;6:38753. doi: 10.1038/srep38753 (PMC5144063; doi:10.1038/srep38753)
Supplement: Supplementary Information [file srep38753-s1.pdf]

Supplementary information

Inhibition of PKR protects against H_2O_2-induced injury on neonatal cardiac myocytes by attenuating apoptosis and inflammation

Yongyi Wang[1], Min Men[2], Bo Xie[1], Jianggui Shan[1], Chengxi Wang[1], Jidong Liu[1], Hui Zheng[1], Wengang Yang[1], Song Xue[&1], Changfa Guo[&3]

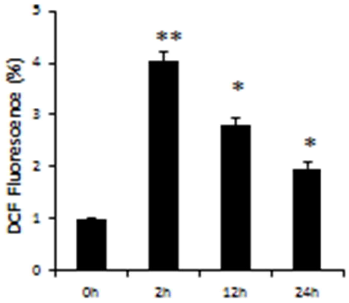

Supple Fig.1 Time course analyses of intracellular ROS after H_2O_2 stimulation. Cells were incubated with 50μM H_2O_2. Fluorescence was measured at different time intervals as described in materials and methods. Data are presented as mean ± SD (n=3). (** $P<0.01$; * $P<0.05$).

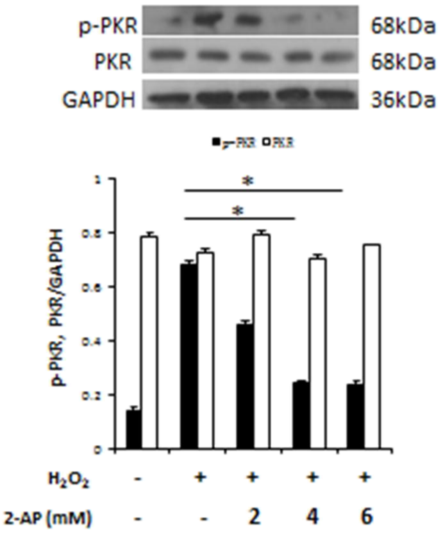

Supple Fig.2 2-AP with different concentrations inhibited PKR activation induced by H_2O_2. Cultured NCM were left untreated or stimulated with 2-AP with different concentrations for 30 minutes, followed by stimulation with H_2O_2 (50μM) for 12h. p-PKR, PKR protein was determined by Western Blotting analysis. (* $P<0.05$).

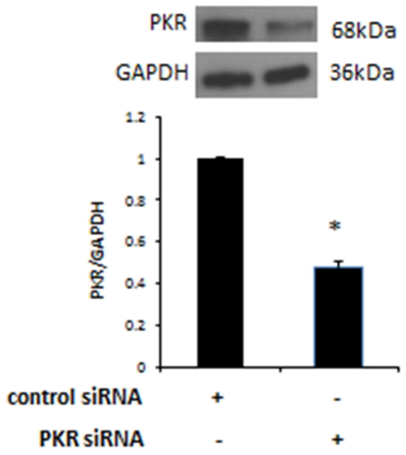

Supple Fig.3 PKR siRNA transfection suppressed PKR expression. Cultured NCM were transfected with control siRNA or PKR siRNA, followed by stimulation with H_2O_2 (50μM) for 12h. PKR protein was determined by Western Blotting analysis. (* $P<0.05$).

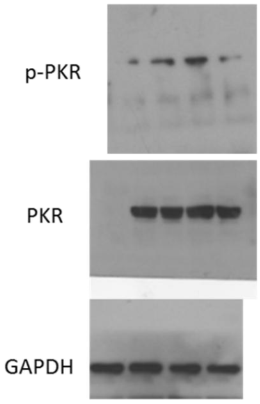

Original images for Figure1 A

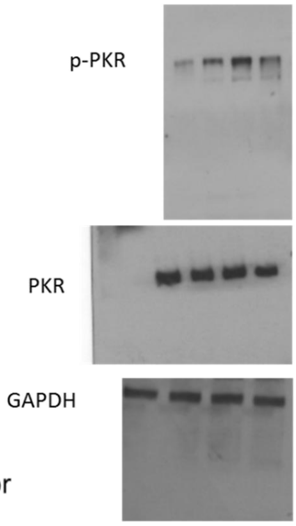

Original images for Figure1 B

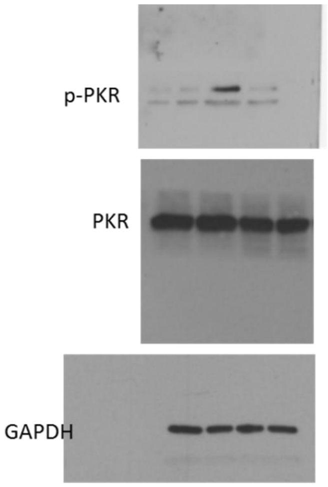

Original images for Figure1 C

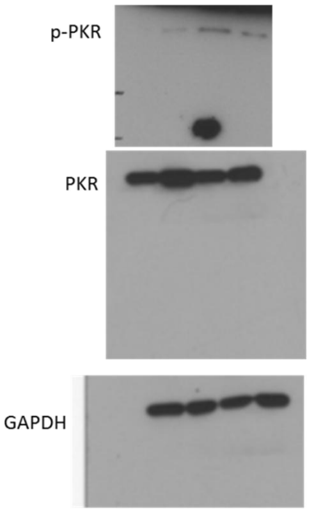

Original images for Figure1 D

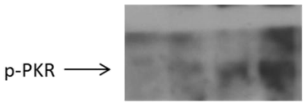

Original figures for Figure1 E

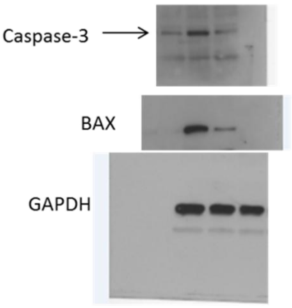

Original images for Figure 3C

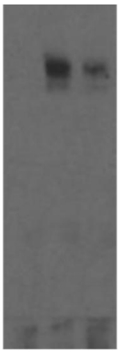

Original images for Figure 4A

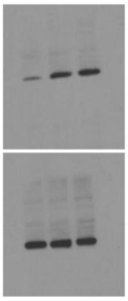

Original images for Figure 5A

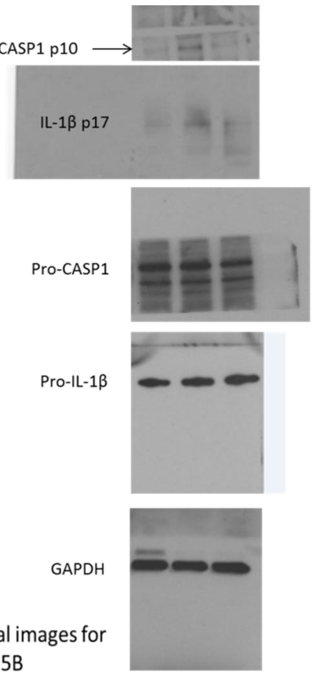

Original images for Figure 5B

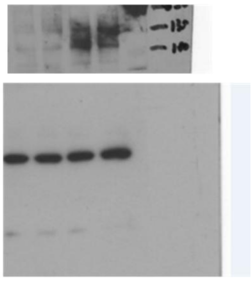

Original images for Figure 6B

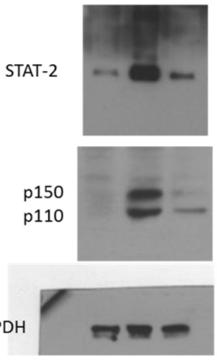

Original images for Figure 6C

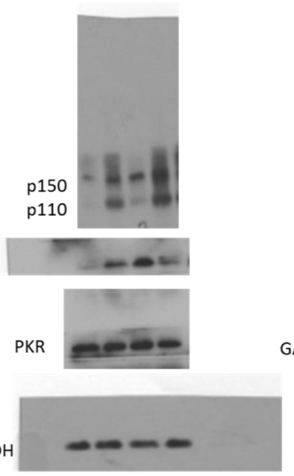

Original images for Figure 6D

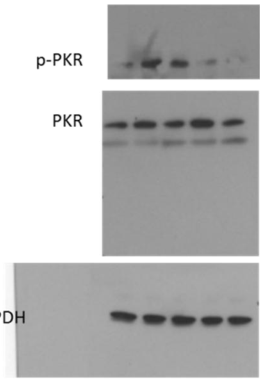

Original images for Supple Figure 2

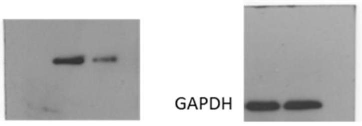

Original images for Supple Figure 3

Supple Fig.4 Full length blots.
